# Isolated sleep paralysis

**DOI:** 10.4103/0019-5545.43064

**Published:** 2005

**Authors:** Neena S. Sawant, Shubhangi R. Parkar, Ravindra Tambe

**Affiliations:** * Associate Professor, Department of Psychiatry, Seth G.S. Medical College and K.E.M. Hospital, Parel, Mumbai 400012; ** Professor and Head, Department of Psychiatry, Seth G.S. Medical College and K.E.M. Hospital, Parel, Mumbai 400012; *** Ex-Resident, Department of Psychiatry, Seth G.S. Medical College and K.E.M. Hospital, Parel, Mumbai 400012

**Keywords:** Sleep paralysis, isolated sleep paralysis

## Abstract

Sleep paralysis (SP) is a cardinal symptom of narcolepsy. However, little is available in the literature about isolated sleep paralysis. This report discusses the case of a patient with isolated sleep paralysis who progressed from mild to severe SP over 8 years. He also restarted drinking alcohol to be able to fall asleep and allay his anxiety symptoms. The patient was taught relaxation techniques and he showed complete remission of the symptoms of SP on follow up after 8 months.

## INTRODUCTION

Sleep paralysis (SP) is a transient and generalized inability to move and speak which occurs during the transition period between sleep and wakefulness with accompanying hypno-gogic or hypnopompic hallucinations that make the episodes extremely distressing. Wilson in 1928 introduced the term ‘sleep paralysis’ to describe attacks of powerlessness that can occur upon awakening.^1^

Sleep paralysis has received some attention in the literature on narcolepsy, as an auxiliary symptom of this disorder. However, it can also occur independently. SP occurs in an estimated 30%–50% of patients with narcolepsy.^2^ The International Classification of Sleep Disorders (ICSD) (1990)^3^ has proposed the following grading of severity for SP:

- Severe SP – Episodes of SP occurring at least once per week
- Moderate SP – Episodes of SP occurring at least once a month but less than once a week
- Mild SP – One or more episodes less than once a month or occurrence of the last episode more than 1 year earlier
- No SP – No symptoms of SP

For severe and moderate SP, the last episode must have occurred within the year. The prevalence of isolated SP in the general population is, however, not well documented. Several studies have described psychological stress, post-traumatic stress disorder or panic attacks to be contributing to SP.^4^,^5^ Factors such as fatigue, irregular life patterns and sleep deprivation may also predispose individuals to SP.^6^

Sleep paralysis tends to occur more commonly in association with visual hallucinations which may be structured, unstructured or bizzare. The sense of a presence in the room is common, as is the visual experience of actually seeing someone in the room. Auditory hallucinations may be in the form of a loud buzzing sound or as the sound of the wind or waves. A sense of suffocation, heaviness of the chest or a choking sensation are frequent somatic experiences.^2^,^7^ It is important to recognize that the fear and anxiety associated with SP appear to be triggered by the symptom of being unable to move or shout.

A case of severe, isolated sleep paralysis is reported here.

## THE CASE

A 35-year-old married man came to the de-addiction centre for the first time for treatment. The patient had been consuming alcohol for 10 years, and had a history of tolerance and withdrawal symptoms. He gave a history of multiple periods of abstinence, the last abstinent period being the maximum (3 years). He relapsed just 6 months before the current psychiatric referral. He also gave a history of panic attacks for the past 5 years, which had increased in frequency over the past 6 months. As the patient complained that the panic symptoms occurred during sleep, the symptoms were evaluated. It was then that the history of SP became evident. The patient described all the episodes that had occurred during sleep, in which he would see an unknown woman standing by his bedside, threatening to kill him. He also felt as if she was trying to throttle him or restrain his limbs. At that time he would find himself completely unable to move his limbs or even shout or call for help. These episodes of powerlessness would last for 2–5 minutes leaving the patient extremely frightened. The episodes had started 8 years ago, and occurred about twice or thrice a year but were not found to be distressing by the patient at that time. However, since the past 6 months, the frequency of the episodes had increased to about twice a week and this resulted in insomnia. The patient also started having palpitations, a panicky feeling, a fear that something bad would happen and anticipatory anxiety about the attack. He would then be unable to sleep the entire night and, to allay the anxiety and get some sleep, he restarted drinking alcohol. He had been abstinent for 3 years and, though these attacks had also occurred during the abstinence period, he had not relapsed. This time, in view of the severity of the symptoms and the increased frequency, he considered himself helpless. His consumption of alcohol would be one-quarter at night which then increased to one-quarter in the day too, due to his withdrawal symptoms. Despite this, he did not get relief from the sleep-related attacks. Due to the nature of the symptoms, the patient and his family had also gone to several faith healers without any relief. A psychiatrist was considered the last resort. Apart from the cultural belief of black magic there was no history suggestive of psychosis, mania or depression in the patient. No cataplectic attacks or daytime drowsiness was reported. None of the episodes had been precipitated by any obvious emotional stressors. There was no family history of SP or any psychiatric illness.

On examination, the patient was conscious, his pulse was 80/minute, the BP was 120/80 mmHg, the pupils were bilaterally equal and reacting to light. General and systemic examinations were normal and no focal neurological deficit was seen. Fundoscopy was normal.

### Investigations

An EEG scan done in the intervening period did not reveal any abnormality. Blood biochemistry and serology were normal and he was HIV negative. The X-ray of the chest was also normal.

*Psychological tests:* The IQ on WAPIS was 101, and the Rorschach profile was invalid. The Minnesota Multiphasic Personality Inventory (MMPI) showed elevation on scale 8,9 suggesting a person with the code who spends a great deal of time in fantasy and daydreams.

In the ward, the patient was detoxified and his alcohol withdrawal symptoms were treated with Tab. lorazepam 8–10 mg/day in divided doses for 5–7 days with vitamin supplements. He continued to have SP in the ward. After the withdrawal symptoms had subsided, he was started on Tab. zolpidem, a sedative–hypnotic at a dose of 10 mg at bedtime to help him sleep as well as decrease the anxiety. This was given for 3 weeks in the ward and on discharge, the dose was tapered off to 5 mg at bedtime for the next 2 weeks and then stopped on follow up. He was also taught relaxation techniques for his panic and told about sleep hygiene. The patient has had a complete remission of the symptoms of SP as well as the panic attacks on a follow up of 8 months though he continues to consume alcohol on and off.

## DISCUSSION

This patient had symptoms of isolated SP for 8 years. Various diagnoses such as narcolepsy, panic disorder and psychosis were considered. However, he did not satisfy the criteria for narcolepsy, though the literature suggests that SP is linked to narcolepsy. Hence, it could still be considered as a variant of narcolepsy. The true prevalence of SP in the general population remains unknown. Our patient had developed anticipatory anxiety before falling off to sleep as he was petrified of the powerless-ness occurring during the episode and his state of immobility. Though 5%–10% of patients with panic disorder may have sleep-related panic attacks with generalized muscular atony as a consequence of autonomic dysregulation during the transition from stage 2 to stage 3 sleep, our patient also had hypnogogic/hypnopompic hallucinations which are not a feature of classical panic disorder. The patient expressed intense fear relating to the episode, leading to insomnia and the criteria for schizophrenia were not met with. The symptoms were severe enough to cause a relapse of his previously achieved abstinent state as the patient restarted alcohol consumption for relief of his symptoms. The patient had progressed from having mild SP for over 8 years to severe SP over the past 6 months. Severe SP is known to be associated with bipolar and anxiety disorders, whereas depressive disorders are more common in patients with mild SP.^5^ Studies have reported a higher prevalence of isolated SP in the fifth and sixth decades of life, more in females and the widowed.^2^

The treatment proposed for isolated SP falls into two categories: psychological and physiological. Several authors have reported psychoanalytical treatment for the resolution of sleep paralysis.^2^,^7^ Simple reassurance may also help. Auto-hypnosis has also been tried. Drugs used in the treatment of SP include amitriptyline (75–100 mg) in association with l-tryptophan (2–4 g) at bedtime.^8^ We considered starting our patient on amitriptyline but as he was better with relaxation therapy on follow up, we did not put him on any medication.

SP may reflect various causes, it could be induced by lifestyle factors such as sleep deprivation or may be secondary to psychiatric or sleep disorders. After all the possible causes are ruled out, the prevalence of SP is only 1.7% in the population. Polysomnography with EMG studies would be useful in supporting a diagnosis of SP, which could not be done in this patient. Thus, a better understanding of the identifiable and treatable causes of SP, and of the isolated type would help us in better managing these patients.

## References

[1] Aldrich MS, Kryger MH, Roth T, Dement C (1994). Cardinal manifestation of sleep disorders. Principles and practice of sleep medicine.

[2] Goode GB (1962). Sleep paralysis. Arch Neurol.

[3] American Sleep Disorders Association (1990). International Classification of Sleep Disorders (ICSD). Diagnostic and coding manual.

[4] Bell CC, Dixie-Bell DD, Thompson B (1986). Panic attacks: Relationship to isolated sleep paralysis. Am J Psychiatry.

[5] Ohayon MM, Zulley J, Guilleminault C (1999). Prevalence and pathologic associations of sleep paralysis in the general population. Neurology.

[6] Fukuda K, Miyasita A, Inugami M (1987). High prevalence of isolated sleep paralysis: Kanashibari phenomenon in Japan. Sleep.

[7] Liddon SC (1967). Sleep paralysis and hypnagogic hallucinations. Their relationship to the nightmare. Arch Gen Psychiatry.

[8] Roth B, Williams RL, Karacan I (1978). Narcolepsy and hypersomnia. Sleep disorders: Diagnosis and treatment.

